# Mechanical Intestinal Obstruction in a Porcine Model: Effects of Intra-Abdominal Hypertension. A Preliminary Study

**DOI:** 10.1371/journal.pone.0148058

**Published:** 2016-02-05

**Authors:** L. Correa-Martín, E. Párraga, F. M. Sánchez-Margallo, R. Latorre, O. López-Albors, R. Wise, M. L. N. G. Malbrain, G. Castellanos

**Affiliations:** 1 Laparoscopy Department, Jesús Usón Minimally Invasive Surgery Centre (JUMISC), Cáceres, Spain; 2 Department of Anatomy and Comparative Pathology, Veterinary Faculty, University of Murcia, Murcia, Spain; 3 Critical Care Unit, Edendale Hospital, Pietermaritzburg, South Africa, and Department of Anaesthetics and Critical Care, Perioperative Research Group, Nelson R Mandela School of Medicine, University of KwaZulu-Natal, Durban, South Africa; 4 Medical and surgical ICU and high care Burn Unit, Ziekenhuis Netwerk Antwerpen, Antwerpen, Belgium; 5 Department of General Surgery, Virgen de la Arrixaca General University Hospital, Murcia, Spain; The First Affiliated Hospital of Nanjing Medical University, CHINA

## Abstract

**Introduction:**

Mechanical intestinal obstruction is a disorder associated with intra-abdominal hypertension and abdominal compartment syndrome. As the large intestine intraluminal and intra-abdominal pressures are increased, so the patient’s risk for intestinal ischaemia. Previous studies have focused on hypoperfusion and bacterial translocation without considering the concomitant effect of intra-abdominal hypertension. The objective of this study was to design and evaluate a mechanical intestinal obstruction model in pigs similar to the human pathophysiology.

**Materials and Methods:**

Fifteen pigs were divided into three groups: a control group (n = 5) and two groups of 5 pigs with intra-abdominal hypertension induced by mechanical intestinal obstruction. The intra-abdominal pressures of 20 mmHg were maintained for 2 and 5 hours respectively. Hemodynamic, respiratory and gastric intramucosal pH values, as well as blood tests were recorded every 30 min.

**Results:**

Significant differences between the control and mechanical intestinal obstruction groups were noted. The mean arterial pressure, cardiac index, dynamic pulmonary compliance and abdominal perfusion pressure decreased. The systemic vascular resistance index, central venous pressure, pulse pressure variation, airway resistance and lactate increased within 2 hours from starting intra-abdominal hypertension (p<0.05). In addition, we observed increased values for the peak and plateau airway pressures, and low values of gastric intramucosal pH in the mechanical intestinal obstruction groups that were significant after 3 hours.

**Conclusion:**

The mechanical intestinal obstruction model appears to adequately simulate the pathophysiology of intestinal obstruction that occurs in humans. Monitoring abdominal perfusion pressure, dynamic pulmonary compliance, gastric intramucosal pH and lactate values may provide insight in predicting the effects on endorgan function in patients with mechanical intestinal obstruction.

## Introduction

Mechanical intestinal obstruction (MIO) is a common presenting problem in emergency departments [1, 2]. A frequent cause, in the small intestine, is the presence of adhesions. However, approximately one third of cases of acute MIO are caused by tumors and volvulus located in the large intestine [1, 3, 4]. Clinical findings depend on several factors, including the competency of the ileocaecal valve. This situation may lead to increased intraluminal pressure in the colonic segment between the ileocaecal valve and the site of obstruction, resulting in increased intra-abdominal pressure (IAP) and a greater risk of intestinal ischemia and subsequent perforation [4, 5]. The development of intra-abdominal hypertension (IAH), defined as a consistent increase in IAP equal to or greater than 12 mmHg, and a sustained increase in IAP above 20 mmHg, may lead to abdominal compartment syndrome (ACS), multiple organ failure and subsequently a poor outcome [6–8]. The World Society on Abdominal Compartment Syndrome (WSACS, www.wsacs.org) recently updated the 2006 consensus definitions and guidelines [9–11].

Intestinal problems, such as abdominal surgery, ileus and abdominal distension have previously been listed as risk factors for IAH and ACS [12]. A substantial number of patients with marked intestinal distension, due to caecal and colonic dilatation usually have a complete colonic obstruction with a competent ileocecal valve [4]. Previous studies have shown that increased IAP is a major role-player in the development of morbidity related to intestinal occlusion [6]. Most experimental studies on intestinal obstruction [13–18], have to date been conducted in rats, focusing on the evaluation of the effects of ischemic hypoperfusion and bacterial translocation. These studies simulated intestinal obstruction by restricting blood supply to the intestine using ligation at the level of the ileocaecal valve [13, 15–18] or using materials to reduce the intestinal lumen [14]. These laboratory models of obstructions were maintained for 12 to 72 hours either with or without direct restriction of the splanchnic vasculature. Although hypoperfusion is an important consequence of an obstructive problem, previous studies have not considered the increase in IAP that accompanies this process, and thus only partially reflect the real clinical situation. Given the lack of available research in the setting of intestinal obstruction in combination with increased IAP, we aimed to design an animal model to simulate the scenario encountered in clinical practice. We wanted to assess the pathophysiological effects on endorgan function related to IAH that would accompany MIO. As others have suggested, the use of a porcine model is ideal since its hemodynamic, ventilatory and physiological features are similar to humans, whilst it also allows for better monitoring than smaller animals [19–22].

The primary objective of this study was to design and validate an experimental pig model of MIO and IAH under similar conditions to those described in the clinical setting.

## Materials and Methods

Fifteen Large White female pigs (23.4 ± 3.7 kg) from the animal facility of the Minimally Invasive Surgery Center Jesus Usón, Cáceres (CCMIJU), Spain were studied. This study was carried out in strict accordance with the recommendations in the Royal Decree 1201/2005 of 10 October 2005 (BOE from Oct. 21) on protection of animals used for experimentation and other scientific purposes. All experimental protocols were approved by the Committee on the Ethics of Animal Experiments of Minimally Invasive Surgery Centre Jesús Usón and by the Council of Agriculture and Rural Development of the Regional Government of Extremadura.

After 24 hours of fasting, the animals received intramuscular premedication, consisting of atropine (0.04 mg/kg), diazepam (0.4 mg/kg) and ketamine (10 mg/kg). The animals were preoxygenated with a FiO_2_ of 1.0 (fresh gas flow of 3–5 l/min), before the administration of propofol 1% (3mg/kg), after which they were intubated and mechanically ventilated. Anesthesia was maintained with isofluorane (MAC of 1.25) and 0.9% sodium chloride intravenous fluids (2ml/kg/h). In addition, intraoperative analgesia was provided with an infusion of remifentanil (0.3 ug/kg/min). On completion of the study, the animals were euthanized following the guidelines of the American Veterinary Medical Association Panel on Euthanasia [23] using potassium chloride (KCl, 1-2mmol/kg).

### Study design

**Fig 1:**
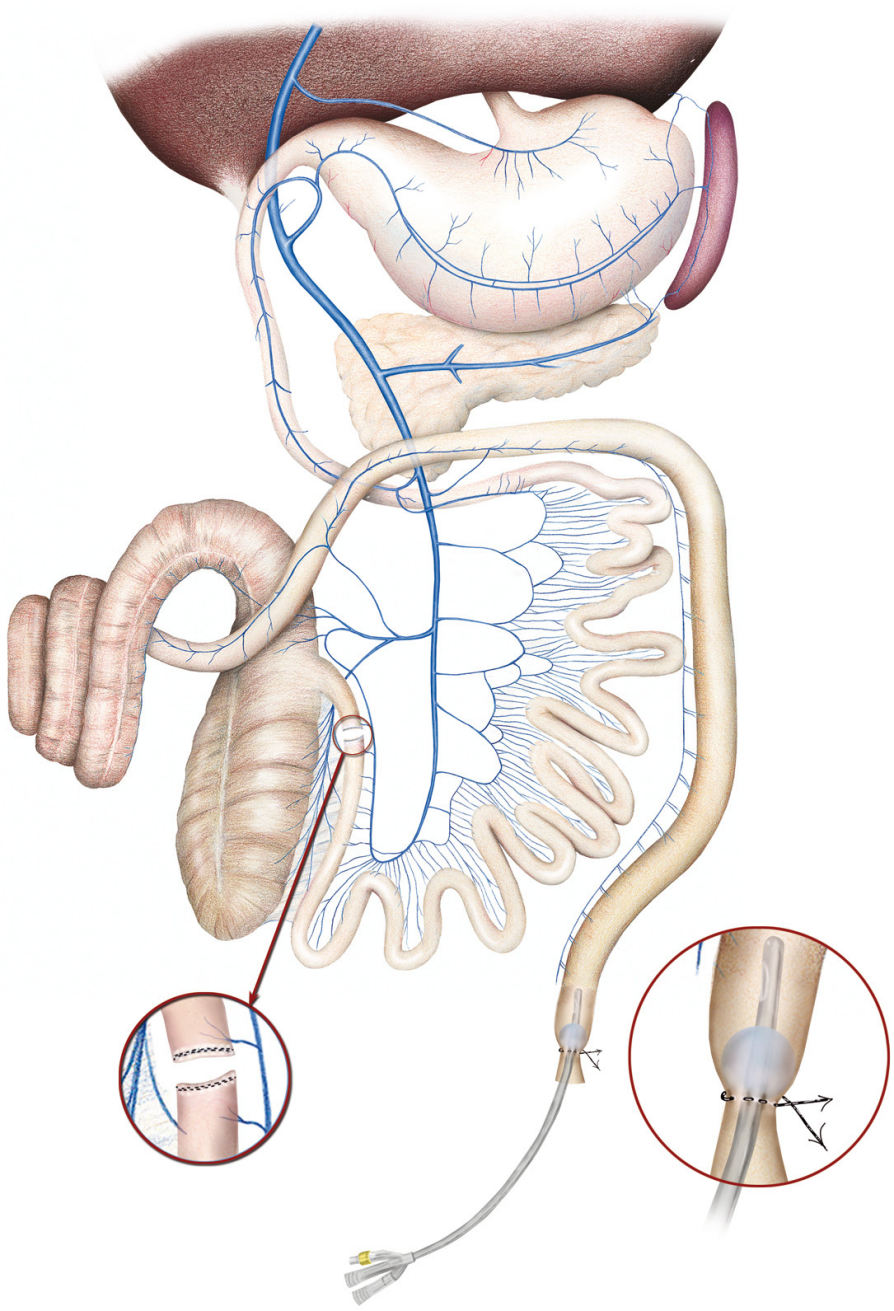
Schematic diagram of the surgical procedure to achieve mechanical intestinal obstruction.

**Fig 2:**
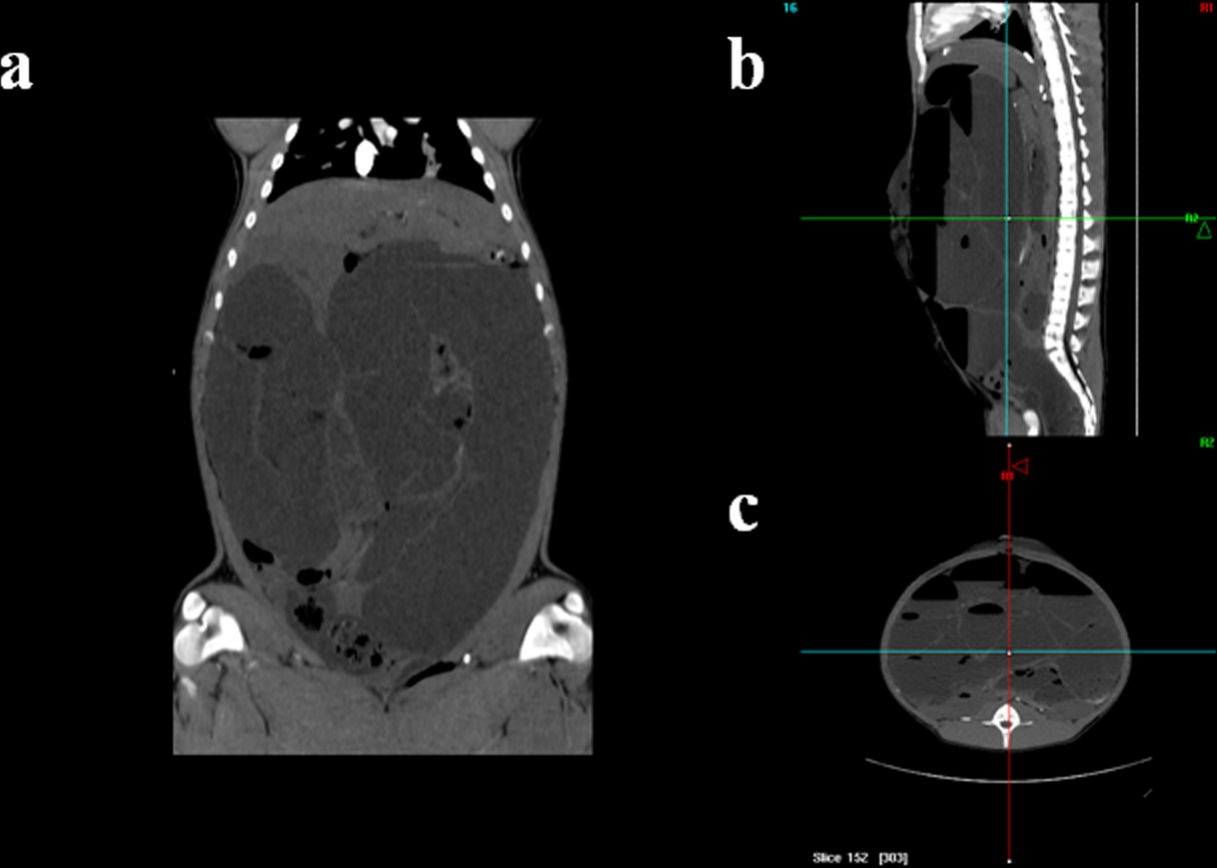
**a.** CT image in frontal plane of the pig abdomen. **b.** CT image in sagittal plane of the pig abdomen. **c.** CT image in transversal plane of the pig caudal abdomen.

Three groups were established: One control (C, n = 5) and two IAH experimental groups produced by MIO. The MIO groups were maintained for either 2 hours (*Experiment 1*, n = 5) or 5hours (*Experiment 2*, n = 5). In the MIO groups, are in forced laparoscopic suture at the ileocaecal valve was placed in order to achieve mechanical obstruction (Fig 1). A 0.9% sodium chloride solution was perfused into the colon to simulate IAH. An IAP of 20 mmHg was reached and the pressure was maintained using a 2-way Foley catheter inserted into the rectum. At the end of each experiment, colonic and rectal decompressions were performed. Some animals were also examined by computerized tomography (CT) scan multislice (Philips Brillance 6, Philips Medical System, Best, The Netherlands). An intravenous injection of 2 ml/kg of non-ionic iodinated contrast material (Urografin 76%, Bayer^®^), was administered at a concentration of 270 mg I/mL and a flow rate of 4 mL/s. The images were acquired during a respiratory breath hold. CT scanning was performed at 120 kVp, 250 mAs/slice, 6x0.75-mm collimation, pitch of 0.9, matrix 512 and overlapping increments of 0.5 mm (Fig 2).

#### Experiment 1

The animals IAP were maintained at 20 mmHg for 2 hours. Hemodynamic and respiratory parameters were recorded and blood samples were taken every 30 minutes. Sampling times (T) were initially T1 (after stabilization of IAP at 20 mmHg) and subsequently T2-T5 (every 30 min. from T1) until the end of the experiment (2 hours).

#### Experiment 2

This experiment was conducted on the basis of the preliminary findings following *Experiment 1*. The pigs were kept for 5 hours at an IAP of 20 mmHg and the sampling protocol was the same as for *Experiment 1*, although extended with 30 min. intervals from T6 (150 min.) to T11 (300 min.).

Only the anesthetic procedure was performed in the control group. The same physiological parameters were recorded, and blood samples were taken at the same time intervals as both the MIO groups.

### Data collection

#### IAP measurement

IAP was measured via the direct transperitoneally route, and indirectly via the transvesically route, as described previously [24]. For the transvesical IAP, the urinary catheter was connected to a Foley Manometer (CD Pharma ®, Spain) [25]. The Foley Manometer consisted of a urinary drainage tubing fitted with a bio-filter inserted between the Foley-catheter and the urine drainage bag. The IAP was estimated by the height of the meniscus of the urine column via the bladder,with the reference point at the level where the midaxillary line crosses the iliac crest. The FoleyManometer was scaled in increments of 0.5 mmHg. The direct transperitoneal IAP was continuously measured via a Jackson-Pratt catheter inserted into the abdominal cavity and placed on the liver. This was connected to a pressure transducerand a General Electric Datex-OhmedaS/5TM^®^ compact anesthesia monitor.The system was purged with 0.9% normal saline.

#### Hemodynamic monitoring

Heart rate (HR), mean arterial pressure (MAP), cardiac index (CI), central venous pressure (CVP), pulse pressure variation (PPV) and systemic vascular resistance index (SVRI) were recorded using a 5F thermistor-tipped fiber optic catheter (PV2015L20N; PULSION Medical System®, Munich, Germany) for thermal dye dilution measurement, placed in the descending aorta via the femoral artery [26]. Additional catheters were placed in the caudal cava vein. The SVRI was calculated as Constant x (MAP–CVP)/CI.

#### Respiratory monitoring

Tidal volume (TV), positive end-expiratory pressure (PEEP), peak inspiratory pressure (P_peak_), plateau pressure (P_plat_), pulmonary compliance (C_dyn_ = TV/(P_plat_−PEEP) and airway resistance (R_aw_ = (P_peak_–P_plat_)/Flow) were recorded with a General Electric Datex-OhmedaS/5TM® (Helsinki, Finland) compact anesthesia monitor.

#### Abdominal perfusion pressure (APP) measurement

APP was indirectly calculated from the MAP and IAP according to the formula: APP = MAP- IAP [27].

#### Gastric intramucosal pH (pH_i_), gastric pressure of CO_2_ (P_g_CO_2_) and PCO_2_gap monitoring

Continuous gastric tonometry [28, 29] was monitored using a gastrointestinal catheter (14F Tonometrics ™ Catheter, Datex Ohmeda Tonometrics, Helsinki, Finland) introduced into the stomach. The catheter was connected to a gastric tonometry E-Tone module to estimate pH_i_ from the P_g_CO_2_according to the formula pH_i_ = pH_a_ + LOG (P_a_CO_2_/P_g_CO_2_). ThePCO_2_gap was indirectly calculated from P_g_CO_2_- P_a_CO_2_.

#### Analytical determinations

Blood samples from the femoral vein were obtained for complete blood and biochemical studies (MEK 6318 NIHOM Kohden, Tokyo, Japan). C-reactive protein (CRP), lactic acid and lactate dehydrogenase (LDH) (2300 Metrolab Random Access Clinical Analyzer, Argentina) were determined to assess inflammation and anaerobic metabolism. In addition, arterial blood gas analysis was performed on samples from the common carotid artery to estimate the arterial CO_2_ pressure (P_a_CO_2_) (i- Stat 1 Analyzer, i -Stat cartridge EG6 + Cartridge, Abbott, USA).

### Statistical analysis

A descriptive analysis and analysis of variance (ANOVA) using a general linear model for repeated measures was performed with SPSS 19.0 (SPSS, IBM Statistics Inc., Chicago, IL, USA).Each of the variables was taken as a “within-subjects” factor and the study group (C or MIO) was taken as a “between-subjects” factor. Differences between the groups were analyzed with the Bonferroni test, with significant p values being p < 0.05.

## Results

The experimental study in the MIO groups was accomplished in all except one pig, from *Experiment 2*, that died following colonic perforation.

**Fig 3:**
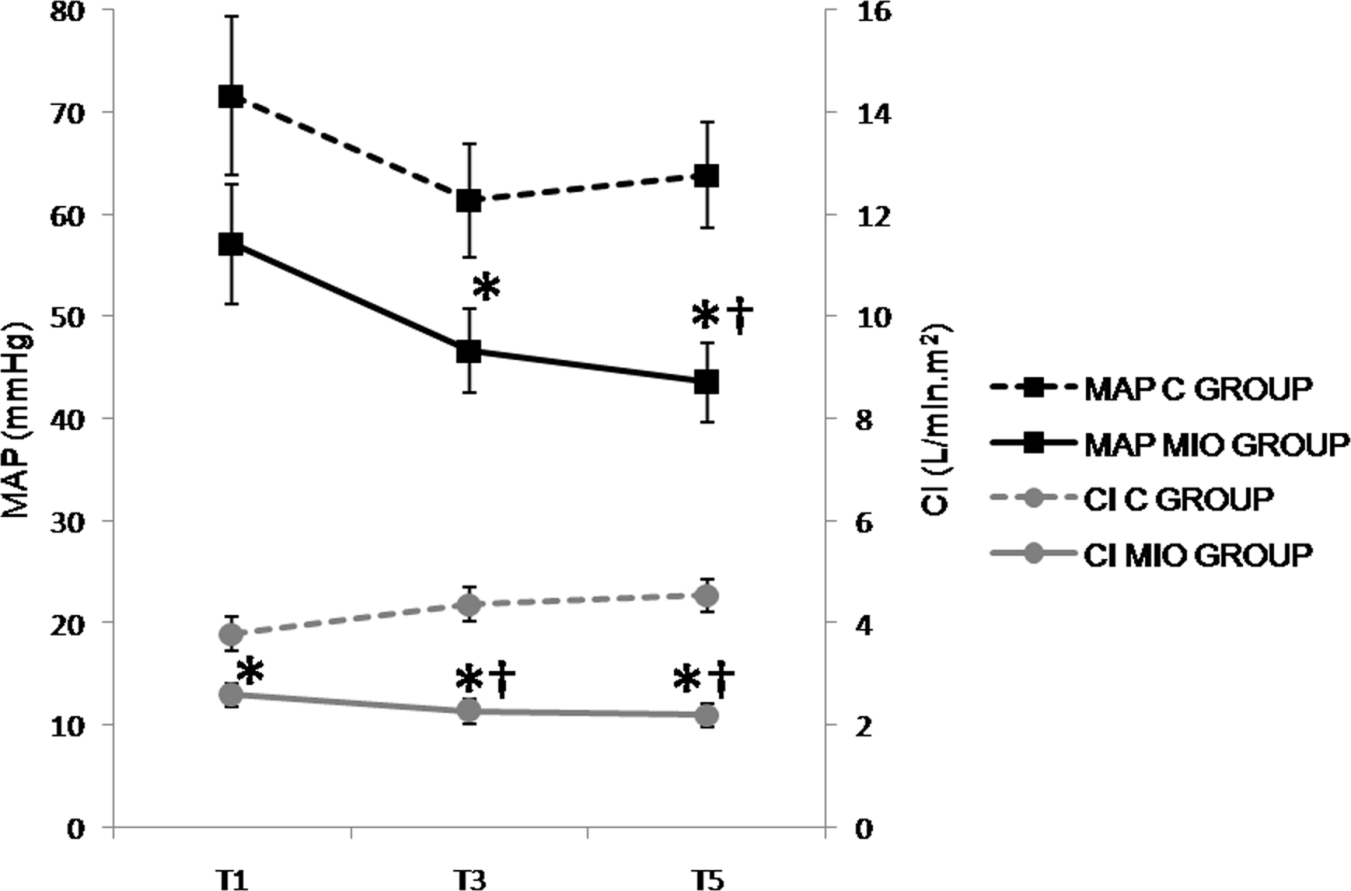
Mean arterial pressure (MAP, mmHg) and cardiac index (CI, L/min.m^2^) values up to2 hours after stabilization of IAH. (*) Indicates significant differences between C and MIO groups at the same sampling interval (p < 0.05). (†) Indicates significant differences in MIO between either T3 or T5 compared to T1 (p < 0.05).

**Fig 4:**
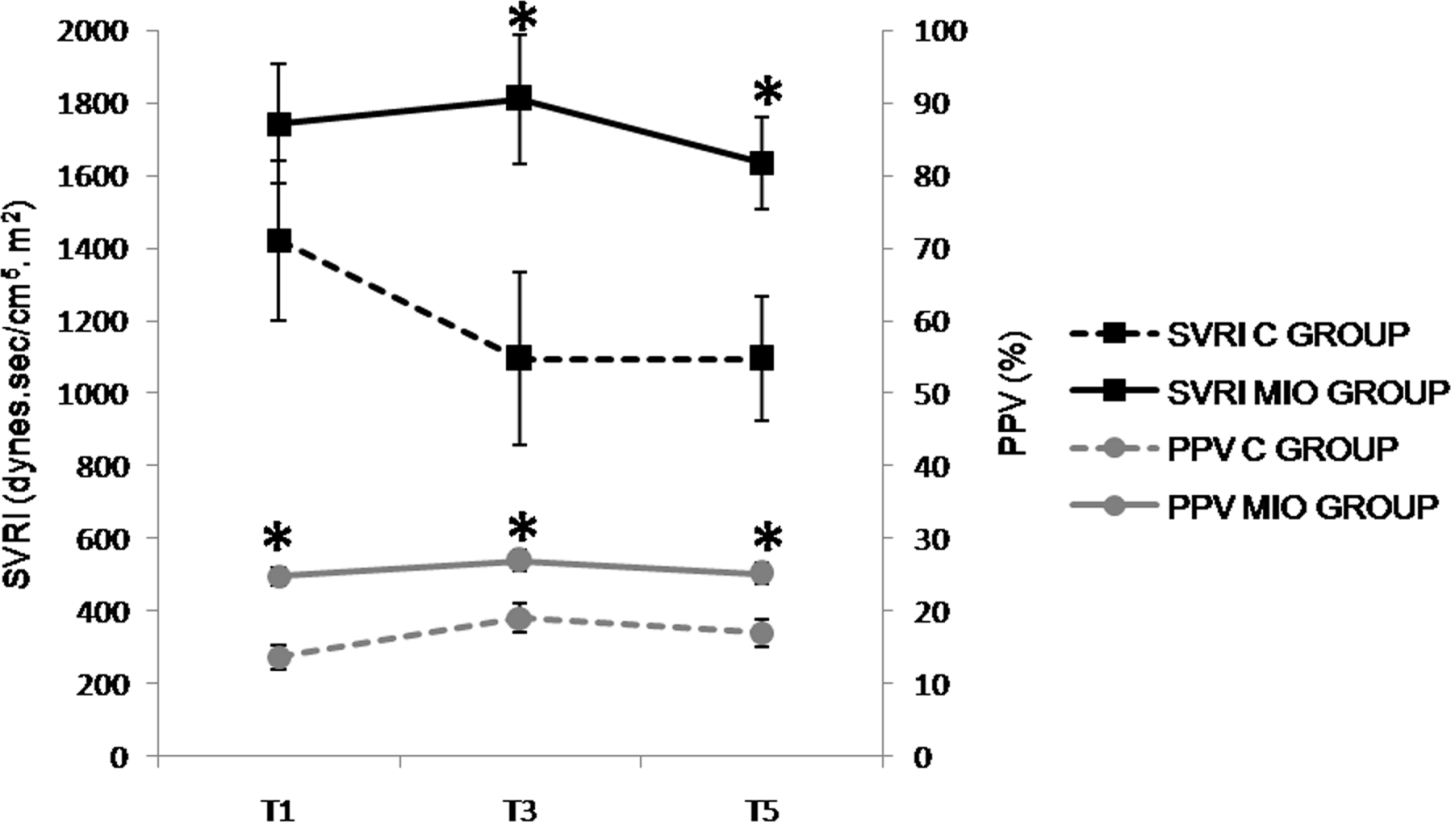
Systemic vascular resistance index (SVRI, dynes.sec/cm^5^.m^2^)and pulse pressure variation (PPV, %) values up to2 hours after stabilization of IAH. (*) Indicates significant differences between C and MIO groups at the sampling interval (p < 0.05).

**Fig 5:**
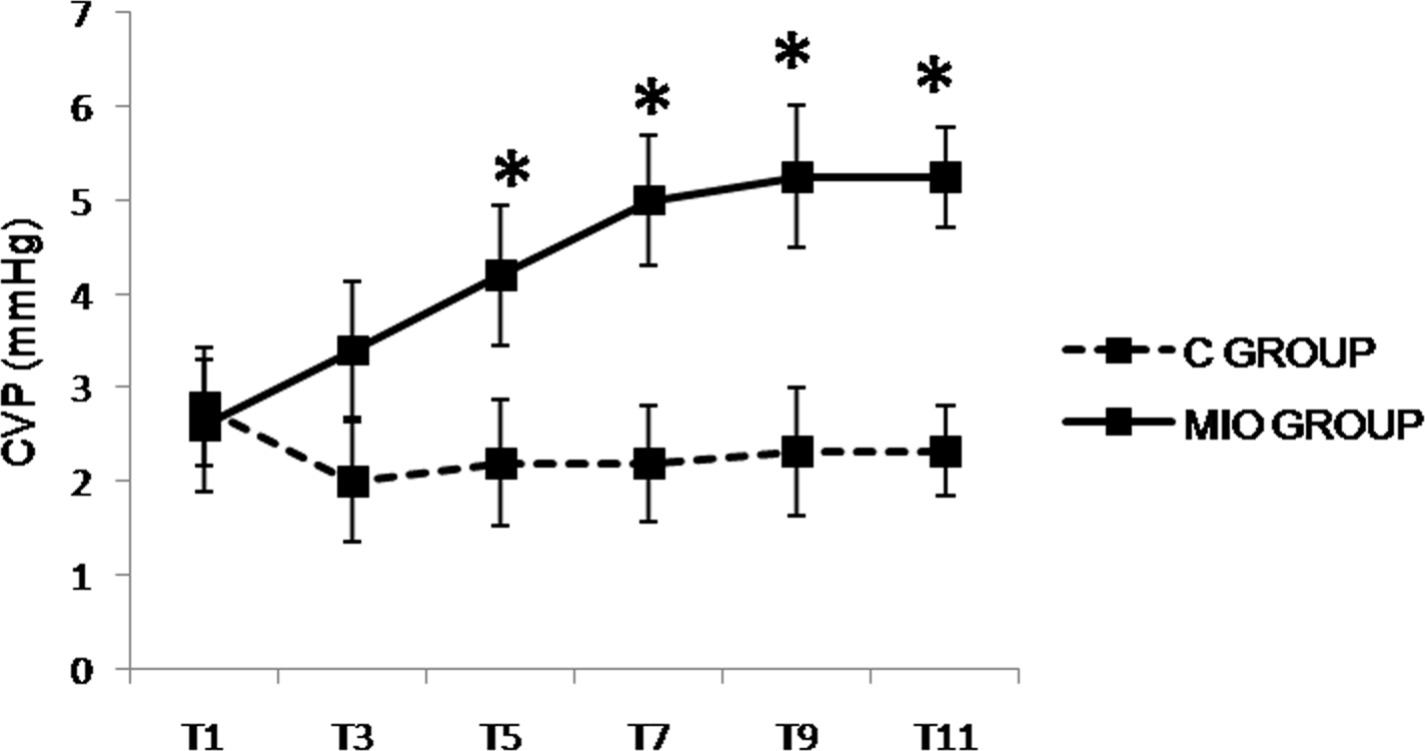
Central venous pressure (CVP, mmHg) values up to 5 hours after IAH stabilization. (*) Indicates significant differences between the C and MIO groups at the same sampling interval (p < 0.05).

**Fig 6:**
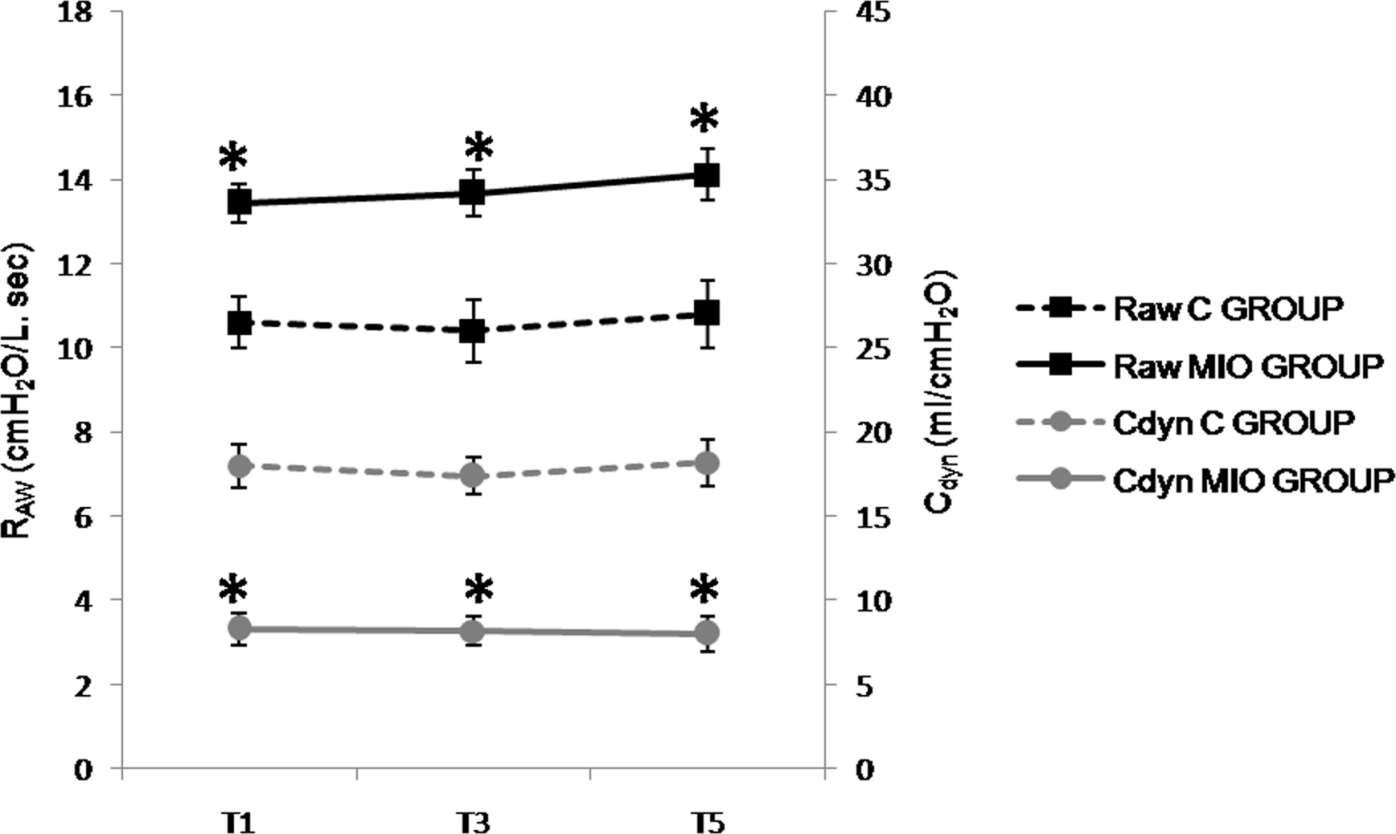
Airway resistance (R_aw_, cmH_2_O/L.sec) and pulmonary compliance (C_dyn_, ml/cmH_2_O) values up to 2 hours after IAH stabilization. (*) Indicates significant differences between the C and MIO groups at the same sampling interval (p < 0.05).

**Fig 7:**
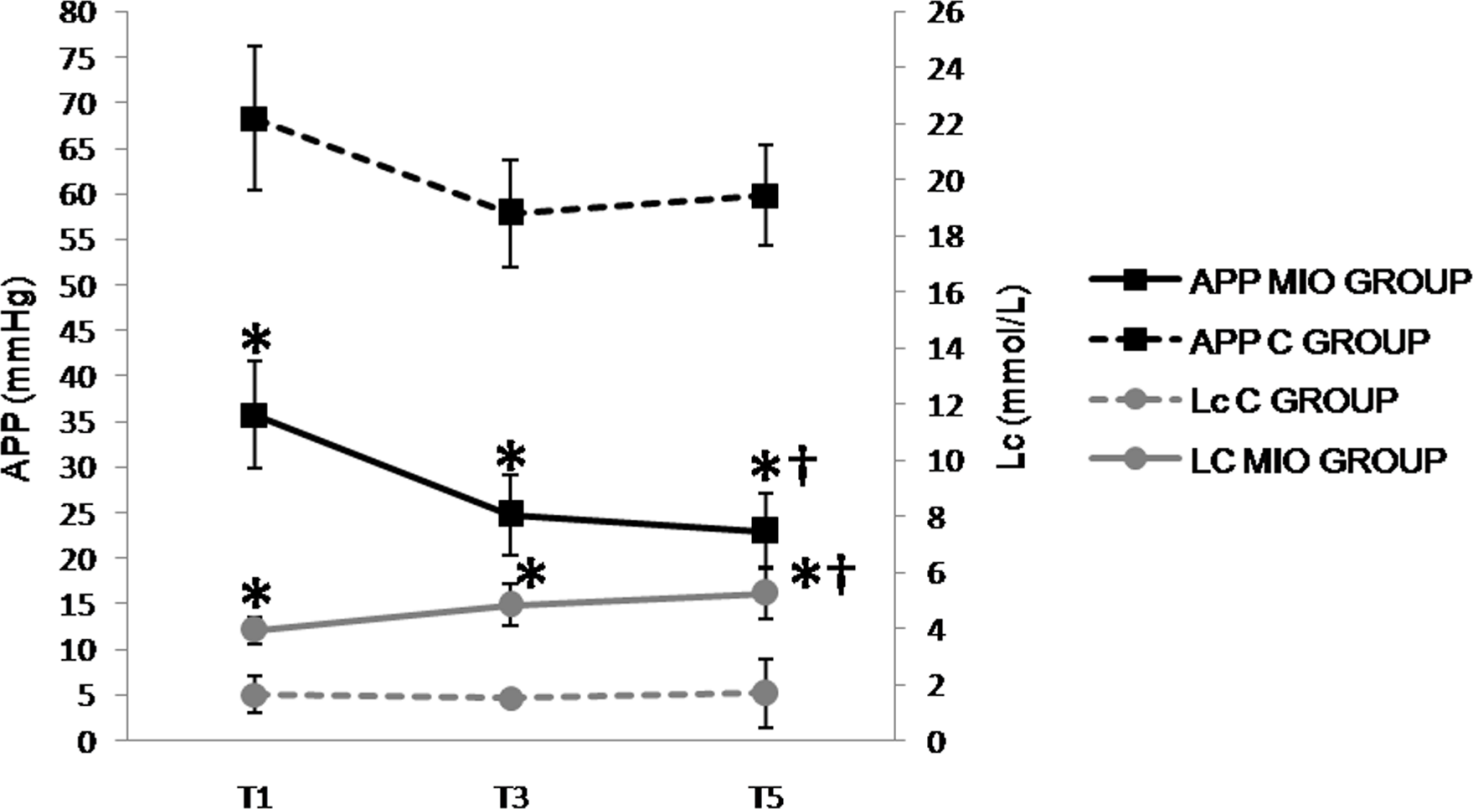
Abdominal perfusion pressure (APP, mmHg) and lactate (Lc, mmol/L) values up to 2 hours after IAH stabilization. (*) Indicates significant differences between the C and MIO groups at the same sampling interval (p < 0.05). (†) Indicates significant differences in MIO between T5 compared to T1 (p < 0.05).

**Fig 8:**
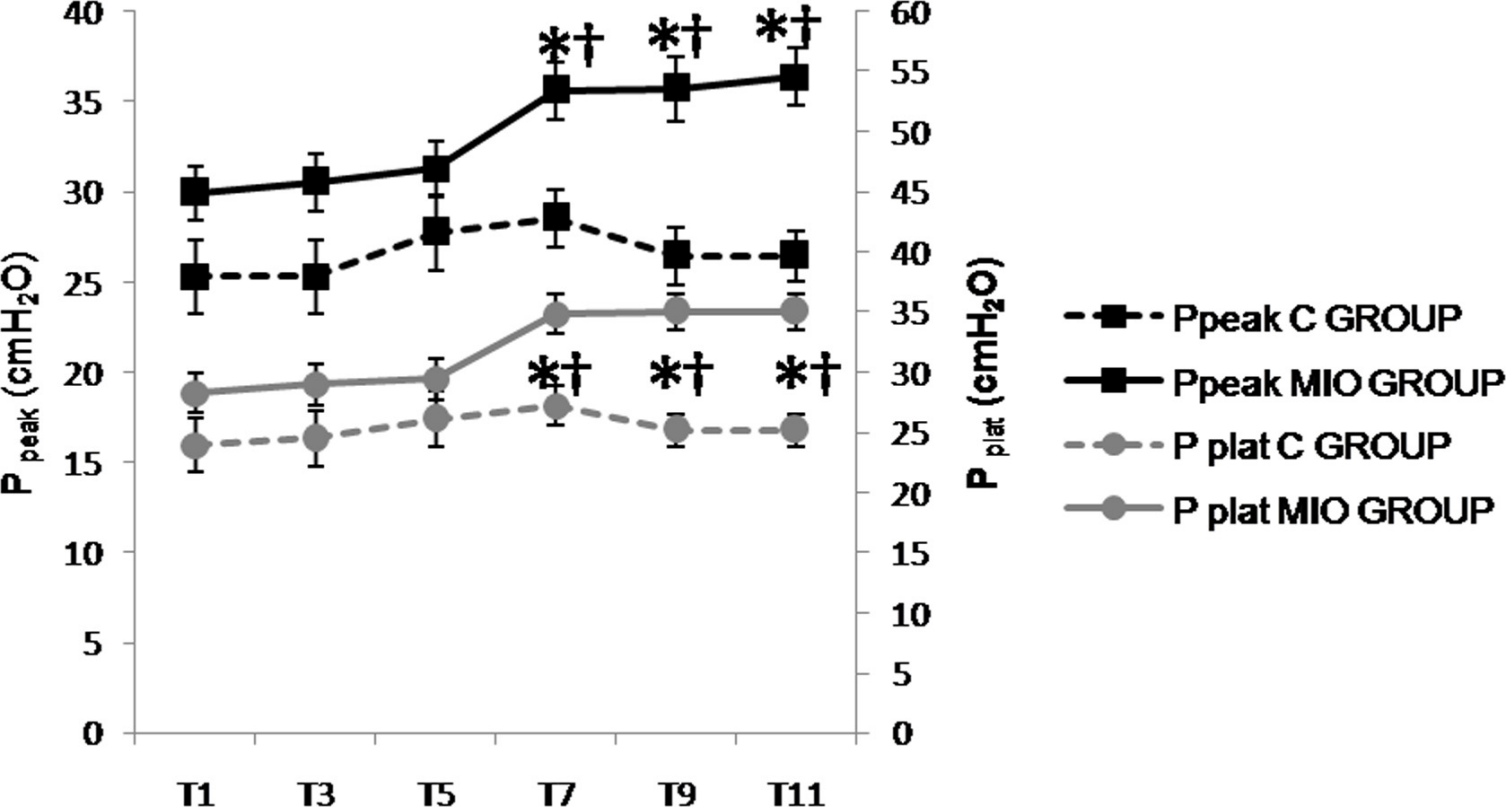
Peak pressure (P_peak_, cmH_2_O) and plateau pressure (P_plat_, cmH_2_O) values up to 5 hours after IAH stabilization. (*) Indicates significant differences between the C and MIO groups at the same sampling interval (p < 0.05). (†) Indicates significant differences in MIO at either T7, T9 or T11 compared to T1 (p < 0.05).

**Fig 9:**
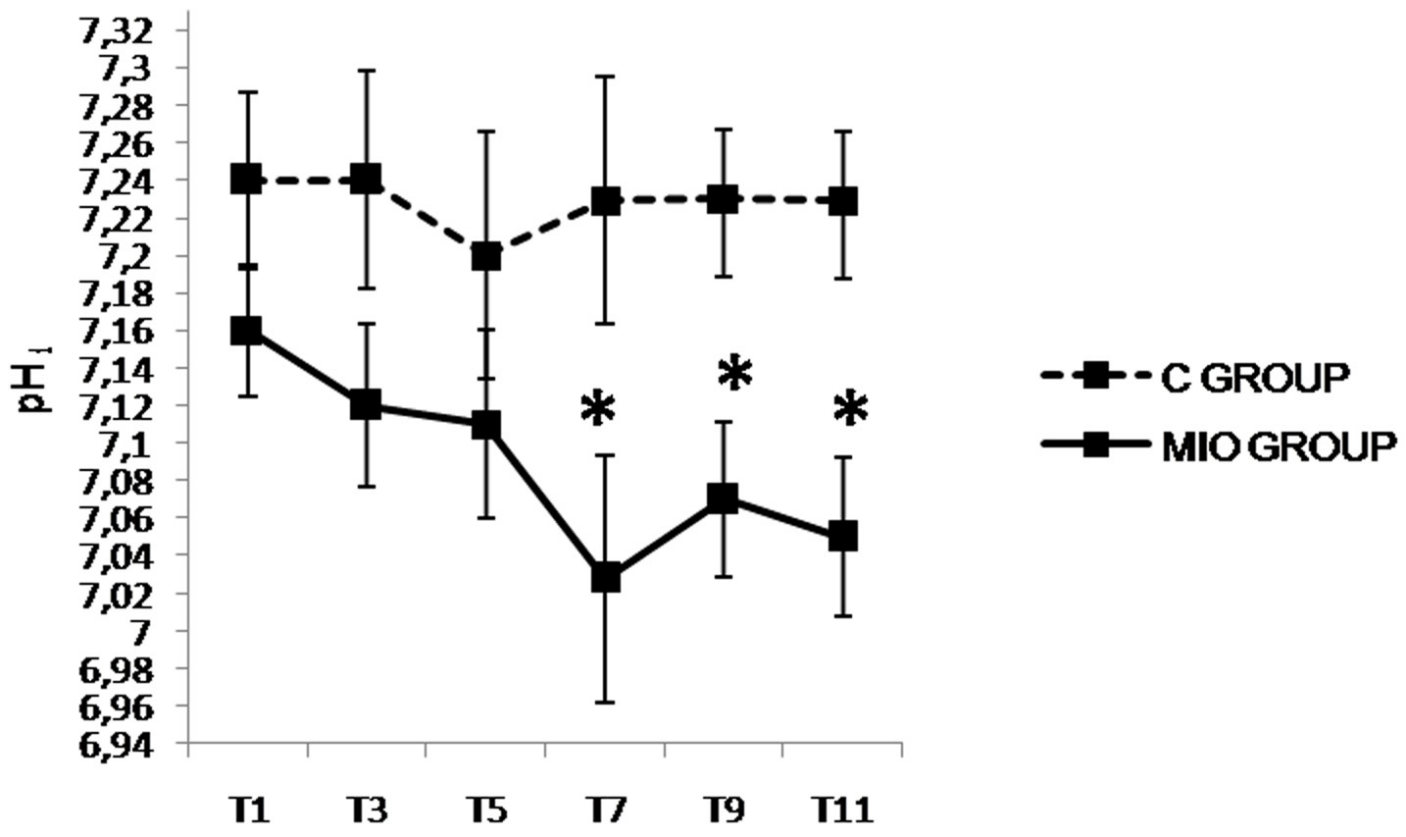
Gastric intramucosal pH (pH_i_) values up to 5 hours after IAH stabilization. (*) Indicates significant differences between the C and MIO groups at the same sampling interval (p < 0.05).

Given that no statistical differences were observed when the sampling intervals were considered for 30 minute intervals (T1-T11), the presentation of the results in Table 1 and Figs 3–9 have been presented with 1-hour intervals for simplification. In more detail, T1, T3 and T5 include results for all the pigs (*Experiments 1* and *2*, n = 9), while T7, T9 and T11 include results only for pigs from *Experiment 2*, due to the longer timeframe for this group intervention (n = 4).

**Table 1:** Cardiorespiratory, biochemical and perfusion parameters obtained in the MIO model until 2 hours.

****CARDIORESPIRATORY VARIABLE****	****T1****	****T3****	****T5****
**HR (bpm)**			
**C**	**92.8 ± 15.8**^**a**^	**101.4 ± 12.4**^**a**^	**109.8 ± 11.3**^**a**^
**MIO**	**125.6 ± 11.8**^**b**^	**103.6 ± 9.3**^**a**^	**96.6 ± 8.4**^**a**^
**P**_**peak**_ **(cmH**_**2**_**O)**			
**C**	**25.3 ± 2**^**ab**^	**25.3 ± 2**^**b**^	**27.7 ± 2**^**a**^
**MIO**	**29.9 ± 1.5**^**a**^	**30.5 ± 1.5**^**a**^	**31.3 ± 1.5**^**a**^
**P**_**plat**_ **(cmH**_**2**_**O)**			
**C**	**23.9 ± 2.2**^**a**^	**24.5 ± 2.3**^**a**^	**26.1 ± 2.3**^**a**^
**MIO**	**28.3 ± 1.6**^**a**^	**29 ± 1.8**^**a**^	**29.5 ± 1.8**^**a**^
**BIOCHEMICAL AND PERFUSION VARIABLE**	**T1**	**T3**	**T5**
**P**_**a**_**CO**_**2**_ **(mmHg)**			
**C**	**43.2 ± 7.4**^**a**^	**45 ± 8.8**^**a**^	**40.5 ± 10**^**a**^
**MIO**	**53.2 ± 5.6**^**a**^	**51.7 ± 6.6**^**a**^	**51.2 ± 7.4**^**a**^
**P**_**g**_**CO**_**2**_ **(mmHg)**			
**C**	**65.9 ± 4.1**^**a**^	**70.5 ± 7.7**^**a**^	**75.2 ± 10.9**^**a**^
**MIO**	**77 ± 3.1**^**a**^	**77.4 ± 5.8**^**a**^	**76.5 ± 8.1**^**a**^
**pH**_**i**_			
**C**	**7.2 ± 0,05**^**a**^	**7.2 ± 0,06**^**a**^	**7.2 ± 0,07**^**a**^
**MIO**	**7.2 ± 0,04**^**a**^	**7.1 ± 0,04**^**a**^	**7.1 ± 0,05**^**a**^
**PCO**_**2**_ **gap (mmHg)**			
**C**	**22.6 ± 5.9**^**a**^	**25.4 ± 8.4**^**a**^	**34.7 ± 9.4**^**a**^
**MIO**	**23.8 ± 8.7**^**a**^	**25.6 ± 10.5**^**a**^	**25.2 ± 11.8**^**a**^
**CRP (mg/L)**			
**C**	**5.6 ± 1.9**^**a**^	**5.5 ± 2.4**^**a**^	**3.96 ± 1.6**^**a**^
**MIO**	**5 ± 1.4**^**a**^	**5.8 ± 1.8**^**a**^	**3 ± 1.2**^**a**^
**LDH (U/L)**			
**C**	**1089.8 ± 167.9**^**a**^	**1047.6 ± 129.4**^**a**^	**1008.8 ± 146.2**^**a**^
**MIO**	**1038.1 ± 125.1**^**a**^	**959.2 ± 96.4**^**a**^	**980.3 ± 108.9**^**a**^

Heart rate (HR, bpm), peak pressure (P_peak,_ cmH_2_O), plateau pressure (P_plat,_ cmH_2_O), arterial CO_2_ pressure (P_a_CO_2,_ mmHg),gastric CO_2_ pressure (P_g_CO_2,_ mmHg), Gastric intramucosal pH (pH_i_), CO_2_ pressure gap (PCO_2_gap, mmHg), lactate dehydrogenase (LDH, U/L), C-reactive protein (CRP, mg/L).

Results are expressed as mean ± SEM. Different superscripts (a, b, ab) in the same row indicate significant differences between sampling times (p < 0.05) in the same group.

### Initial study phase (T1 to T5)

Statistically non-significant results between control and MIO groups are displayed in Table 1 while the most significant results are shown in Figs 3–7. The MIO group in the Fig 5 represents the data of 4 animals due to a failure in data reading.

#### Hemodynamic parameters

The heart rate (HR) decreased from T5 in the MIO group, but there were no significant statistical differences between the experimental groups (Table 1) found. Values for MAP and CI (Fig 3) were lower in the MIO group, with significant differences noted from T3 and T1when compared to the control group, respectively. There was a significant increase in PPV (Fig 4) from T1 in the MIO group in comparison with the control group. The SVRI (Fig 4) and CVP (Fig 5) were significantly higher in the MIO group from T3 and T5 onwards, respectively.

#### Respiratory parameters

P_peak_ and P_plat_ increased in the MIO group (Table 1), although there were no significant differences compared to the control group.A decrease in C_dyn_ and increased R_aw_ were found (Fig 6) with significant differences between the MIO and control groups.

#### Biochemical and perfusion parameters

With the initiation of IAH, there were no significant increases or differences in P_a_CO_2_, P_g_CO_2_ or pH_i_ observed between the MIO and control groups (Table 1).The PCO_2_-gap progressively increased from T1whilethe CRP hardly changed throughout the experiment (Table 1). LDH decreased in the MIO group but not significantly when compared to the control group (Table 1). Lactate values increased and the APP decreased significantly from T1 (Fig 7) in the MIO group as compared to the control group.

### Late study phase (T7 to T11)

The results obtained from the third to fifth hour in hemodynamic, respiratory, laboratory and blood perfusion parameters showed the same trend as during the first two hours (*Experiment1*), with the exception of the P_peak_ and P_plat_ (Fig 8) that increased significantly and pH_i_ (Fig 9) that decreased significantly in the MIO group from T7 when compared to the control group.

## Discussion

The porcine model in our study enabled the simulation of a commonly encountered IAH situation, resulting from MIO due to increased colonic intraluminal pressure, despite anatomical differences in the colon between humans and pigs [30, 31]. Hemodynamic, respiratory and laboratory parameters helped describe the IAH effects on the cardiovascular, pulmonary and renal systems, which are affected when IAP exceeds 18–20 mmHg [32–35].

Hemodynamic changes associated with IAH have previously been well described in humans [32, 33, 36–39]. According to these studies, increased IAP exerts direct pressure on the inferior cava vein and the heart which in turn decreases preload and venous return (while CVP increases due to increased intrathoracic pressures) resulting in a decline in CI [39, 40]. The MAP may initially increase due to blood sequestration from the abdominal vessels (mesenteric capacitance veins), but if sustained, may stabilize and even decrease. Unlike the preload, the after load increases by activation of the renin-angiotensin-aldosterone system causing an increase in the SVRI. In summary IAH reduces cardiac contractility and preload and increases cardiac afterload [40]. In our study, all these effects were observed in the MIO group: CI and MAP decreased (51.4% and 31.7%, respectively), while an increase of 49.4% was obtained in the SVRI 2hours after stabilization of the IAP. These results were consistent with previous studies simulating IAH via fluid instillation into the abdominal cavity [41, 42] or a pneumoperitoneum model [43–47], and showing a decrease in CI and increase in SVRI compared to the baseline. With regard to the MAP, although an initial increase has been described [38, 41], a significant decrease is a common finding in different IAH models [42–49]. In contrast to previous works [42–49] where such a decrease was only observed after several hours of increased IAP, we noticed a drop in MAP just after stabilization of IAH. This may be explained by a greater effect of the MIO model on MAP compared to previously studied models. On the other hand, as was already shown in clinical as well as in other experimental studies [41, 42, 44–47], CVP increases are common during IAH. Our results revealed significant CVP increases from 2 hours after stabilization of the IAP, similar to that observed in others works with higher IAP [44, 45, 47]. This rise, as well as the increased observed in the MAP, was more noticeable in those with an IAP of 25 mmHg [41, 42, 46], indicating a greater effect of the MIO. Our data on functional hemodynamic parameters, such as PPV and SVV, are consistent with previous animal experiments that demonstrated IAH either abolishes or increases threshold values for PPV and SVV to predict fluid responsiveness [50–52]. Using porcine models of simulated IAH, these studies demonstrated that PPV, SVV, as well as caudal cava vein flow fluctuations, in relation to positive pressure ventilation, were dependent on IAP [50]. Therefore, higher thresholds may need to be used among those with IAH to indicate fluid responsiveness [53]. Compared to the normovolemia conditions referred to these previous studies, our results showed increases of around 80% of the PPV at the beginning of IAH.

The respiratory changes seen in clinical studies on intestinal occlusion are thought to be caused by the upward displacement of the diaphragm caused by the increased IAP. The pulmonary C_dyn_ as well as the respiratory rate are usually reduced while the P_peak_, P_plat_ and R_aw_ are increased. Furthermore, atelectasis in the dorsobasal and caudal areas of the lungs has been described because of limited pulmonary inflation and recruitment [33, 36, 37, 54]. In our study an early C_dyn_ decrease of 56% was observed from the onset of IAH, which is consistent with previous work where IAP was increased up to 25 mmHg [41, 42]. In addition, increases in R_aw_, P_peak_ and P_plat_ were observed from the beginning of IAH in our model. Regardless of the experimental model for IAH of 20–25 mmHg [41, 42, 46], significant increases in P_peak_ and P_plat_ have been observed in the period between 1 to 3 hours after commencing the experiment. In addition, those studies where IAP was as high as 30 mmHg after 1 hour of IAH, the P_peak_ and P_plat_ rose by 50% compared to controls [42, 44]. However, in our model these effects only became significant after 3 hours of IAH.

The anaerobic metabolism occurring in patients with IAH results from changes in the cardiorespiratory dynamics that are determined by a P_a_CO_2_ increase and P_a_O_2_ decrease. This may stimulate lactate production, the endpoint of respiratory and metabolic acidosis [55, 56]. In our results P_a_CO_2_ increased by around 25% which is in keeping with the results found by Diaz et al [41] and similar to other work using a porcine model of pneumoperitoneum up to 30 mmHg [45, 47]. In previous IAH models using 20 mmHg and 30 mmHg [43, 44, 46, 48, 49], lactate increases were noted but only became apparent and significant after 3 to 6 hours. Lactate levels in our MIO model increased by up to 5 times compared to the control group before 3 hours.

Several recent reviews on IAH and ACS [33, 36–38, 57] emphasize the negative impact of increased IAP on splanchnic perfusion, which can be significantly decreased in both animal [36, 43, 49, 58, 59] and humans models [9–11, 37, 38]. In relation to this, APP has been suggested as a reliable and easy parameter to assess abdominal perfusion [27, 39, 60] following retrospective analysis which showed it reflected the impact of increased IAP on tissue perfusion better than IAP or MAP alone. Other parameters such as the arterial pH and urine output are not reliable since they may remain within a normal range until the organic dysfunction becomes established. An important result of this study was the demonstration APP being significantly altered soon after IAH stabilization. Compared with the control group, significant decreases in APP of 40% and 61% were observed after the onset and 2 hours of IAH, respectively. This rapid and important deleterious effect on perfusion was consistent with other porcine models at either 20 or 30 mmHg. In ascetic and pneumoperitoneum models of 20 mmHg [48, 49], significant decreases in APP were also observed from the beginning of the study. Furthermore, in models subjected to 30 mmHg [45, 46, 48], significant decreases in APP of 30 to 60% at the beginning were also observed, with further decreases up to 80% at 6 h.

The stomach and intestines are among the most sensitive abdominal organs that express the deleterious effects of IAH. In particular, the use of gastric air tonometry has been established as a useful method to estimate P_g_CO_2_ and pH_i_ [28, 29]. IAH related decreases in gastrointestinal blood flow may cause ischemic events [44, 61],and as a result, pH_i_ may decrease [62] in the early stages of IAH. In this study, pH_i_ only decreased significantly after 3 hours of experimentation. This is not unusual as effects were even more delayed in a pneumoperitoneum model of 20 mmHg [48] where no significant differences were observed up to 5 hours after commencing. However, in two ascetic models with similar IAP, pH_i_ was seriously affected and significantly decreased from the beginning of the study [49] or reduced by more than 4% after 1 hour [42]. Further pH_i_ changes were observed when IAP was increased to 30 mmHg. As a 7% decrease, and absolute values below 7, were recorded after 30 minutes of IAH [42, 48].Thus, the effect of IAP on pH_i_ is influenced by both the method used to achieve the hypertension and the value of abdominal pressure itself. Some clinical studies found an inverse relation between IAP and pH_i_ [62, 63].

The present study has some limitations. The number of animals studied was relatively small and the time course of events was short (up to a maximum of 5 hours). Future studies could try to determine the effects of improved perfusion, including the effects of abdominal decompression, and how this influences endorgan function. Data collection for esophageal pressure, volumetric preload or extravascular lung water, as well as measurements of other perfusion parameters, for example indocyanine green plasma disappearance rate, microdialysis and biomarkers such as citrullin, intestinal fatty acid binding protein, N-Gal or cystatin C, could help to clarify the changes.

In conclusion, we found that the present MIO model affecting the large bowel with a competent ileocaecal valve may be a useful simulation of human intestinal obstruction scenarios. Hemodynamic, respiratory, laboratory and perfusion alterations were similar to those described previously in IAH and ACS studies both in humans and animals, thus supporting the model studied. The most relevant parameters to evaluate the deleterious effects of IAH are monitoring of APP, C_dyn_, pH_i_ and lactate.
